# Use of Thyroid Hormones in Hypothyroid and Euthyroid Patients: A THESIS questionnaire survey of members of the Irish Endocrine Society

**DOI:** 10.1007/s11845-022-03235-z

**Published:** 2022-12-08

**Authors:** Mohamad Mustafa, Elsheikh Ali, Anne McGowan, Laura McCabe, Laszlo Hegedüs, Roberto Attanasio, Endre V. Nagy, Enrico Papini, Petros Perros, Carla Moran

**Affiliations:** 1 Robert Graves Institute, Tallaght University Hospital, Dublin, Ireland; 2 Pharmacy Department, Tallaght University Hospital, Dublin, Ireland; 3 Department of Endocrinology, Odense University Hospital, Odense, Denmark; 4 Scientific Committee Associazione Medici Endocrinologi, Milan, Italy; 5 Division of Endocrinology, Department of Medicine, Faculty of Medicine, University of Debrecen, Debrecen, Hungary; 6 Department of Endocrinology and Metabolism, Opsedale Regina Apostolorum, Rome, Italy; 7 Department of Endocrinology, Royal Victoria Infirmary, Newcastle Upon Tyne, UK; 8 Diabetes & Endocrinology Section, Beacon Hospital, Sandyford, Dublin, Ireland; 9 School of Medicine, University College Dublin, Dublin, Ireland

**Keywords:** Clinical practice, Hypothyroidism, Levothyroxine, Survey, Thyroid hormones

## Abstract

**Background:**

Replacement of thyroid hormones (TH) with Levothyroxine (LT4) is the treatment of choice for hypothyroidism, however, there are aspects of treatment where uncertainties exist and practice varies. Factors influencing initiation and choice of TH replacement may impact patient satisfaction, safety, and health care costs.

**Methods:**

The aim of the study was to examine the attitudes of Irish endocrinologists regarding the treatment of hypothyroid and euthyroid patients with TH. Members of the Irish Endocrine Society (IES) were invited to participate in an online survey.

**Results:**

Forty-eight invitations were sent, and 39 (81.3%) participants responded. All respondents favoured LT4 tablet therapy for treatment of hypothyroidism, but 20.5% prescribed combination therapy (LT4 and liothyronine), and 13% regularly used desiccated thyroid extract. A significant proportion (51%) might prescribe TH in euthyroid patients; 41% for thyroid auto-antibody positive women seeking pregnancy, 18% for goitre and 5% for unexplained fatigue. Many (38%) consider combination therapy in patients with persistent symptoms. Respondents reported seeing LT4 treated patients with persistent symptomatology more frequently and perceive psychosocial factors and comorbidities to be the most common reasons for such symptoms.

**Conclusion:**

LT4 tablets are the treatment of choice for hypothyroidism in Ireland. Approximately a third of Irish endocrinologists either regularly use, or would consider, liothyronine for hypothyroid patients. A significant proportion would give TH to euthyroid individuals in specific circumstances. The prescription of TH amongst Irish endocrinologists was generally in keeping with recommended practice, and areas where practice deviated from guidance were typically where evidence was conflicting or insufficient.

**Supplementary Information:**

The online version contains supplementary material available at 10.1007/s11845-022-03235-z.

## Introduction

Hypothyroidism, either overt or subclinical, is one of the most common endocrine disorders, affecting about 3% of the adult population in Europe [1]. Although presenting features are variable, the majority of patients present with non-specific symptoms such as fatigue, weight gain and constipation. Levothyroxine (LT4) remains the standard treatment for hypothyroidism since it came to market in the 1960s; it is effective, inexpensive, and safe.

A minority of patients with hypothyroidism remain symptomatic following appropriate replacement with LT4 [2] and a myriad of potential causes have been proposed, including the presence of additional, undiagnosed diseases (especially autoimmune), lifestyle factors, psychological morbidity associated with the diagnosis of a chronic condition and others [3–5]. Thyroxine bioavailability can be affected by gastrointestinal conditions causing malabsorption, such as coeliac disease and chronic atrophic gastritis, inability to take thyroxine while fasting, and use of medications such as proton-pump inhibitors, iron supplements, and calcium [6].

Alternative options for thyroid hormone replacement include liothyronine (LT3), combination therapy of LT4 with LT3, and desiccated thyroid extract (DTE). Different LT4 formulations (tablets, liquid solution or soft-gel capsules) are available. Thyroid guidelines globally recommend LT4 as standard therapy [7] however, alternative thyroid hormone replacement options, such as combination therapy with LT4 and LT3, are sometimes considered by patients or physicians, due to the recognition that a subset of patients appropriately replaced with LT4 remain persistently symptomatic [8, 9]. Improved bioavailability of liquid solution and soft-gel capsules compared to tablets has been reported [10–12] however the evidence is not strong and these formulations are more expensive than LT4 tablets, so questions remain regarding their role and cost-effectiveness in managing hypothyroidism [6]. There is insufficient evidence to recommend LT4 and LT3 combination therapy over LT4 monotherapy and there are concerns over possible iatrogenic hyperthyroidism on combination therapy [3].

Residents of Ireland can access the publicly funded national healthcare service, managed by the Health Service Executive (HSE). Hospital care, both inpatient and outpatient, is mainly provided free of charge, but for most people primary care (general practitioner, GP) services incur a cost, although there are exceptions. Prescription charges are subsidised or covered entirely for those on limited income. Almost half of the population (45%) have optional private health insurance. Although there are guidelines regarding use of thyroid function tests in primary care [13] there are no Irish national guidelines on management of hypothyroidism. The majority of patients with hypothyroidism are managed by their GP.

In a report published by the World Health Organization in 2007, the population of Ireland was deemed to have mild iodine deficiency [14], however a later study indicated that 14–15 year-old schoolgirls on the island of Ireland were generally iodine sufficient, but at the lower end of the optimal range (median urinary iodine concentration was 111 μg/L; 100–199 μg/L is sufficient in a non-pregnant population). Only one area, in the west of Ireland, was iodine deficient (median urinary iodine concentration 98 μg/L [15].

Licensed formulations of LT4 in Ireland include tablets and oral solution, unlicensed formulations include LT4 tablets, capsules, oral solutions, suspensions and LT3 tablets and combination LT4/LT3 products (Supplementary Table 1).

Understanding endocrinologists’ prescribing practice in treating hypothyroid patients is essential as it impacts patient satisfaction, safety, and health care costs. We conducted this survey as part of an ongoing international survey of hypothyroidism treatment referred to as THESIS (Treatment of Hypothyroidism in Europe by Specialists: an International Survey), which was developed to evaluate the attitude and practice of European doctors regarding hypothyroidism management.

## Aim

The aim of the study was to examine the attitudes of Irish endocrinologists regarding the treatment of hypothyroid and euthyroid patients with thyroid hormones (TH).

## Methods

### Questionnaire

An identical questionnaire was distributed to all countries participating in THESIS. The questionnaire consisted of 31 questions: 8 questions regarding demographic data (section A) and 23 questions about treating hypothyroid and euthyroid patients (section B, Supplementary Table 2^2^). All responses were anonymous, personal data was protected and due to the survey nature of the study, ethical approval was not required. The questionnaire was initially tested in a pilot study of Italian endocrinologists [16] after translation into Italian, following which it underwent revisions to reach its final form.

### Participant Recruitment

All members of the Irish Endocrine Society were invited to take part by email in July 2020. Those who indicated their agreement to take part were then contacted with a link to complete the survey in September 2020, and weekly reminders were sent to those who did not respond initially. The survey closed in October 2020. All endocrinologists (adult and paediatric, trainee and consultant levels) who were actively working during the time of the study were eligible to participate. Survey responses were anonymously collected and electronically stored on an online platform, Lime Survey, which was accessible by password. Duplicate responses from the same IP-address were blocked.

### Statistical analysis

Data was summarised and analysed using Microsoft Excel and MedCalc statistical software application version 18.5. Percentages were round up to the nearest whole number. Responses were deemed complete if all questions were answered. Where data were missing, the denominator used was the total respondent replies received. Pearson’s x^2^-test was used to test if variables in the demographic data (section A) were independent of the outcome of questions in section B. If any variable was not independent of the outcome in any questions in section B, a logistic regression analysis was done. A two-sided *p* value of < 0.05 was considered statistically significant.

## Results

### Sample characteristics

Respondent characteristics are listed in Table 1. In total, 48 electronic links to the survey were sent, 39 (81%) were returned; 37 of these were complete and 2 were partially completed (2 respondents did not answer question B17, and one of these also omitted one part (subquestion 3) of question B16 (Supplemental Table 2). Gender distribution was similar (female 53%) and most (22/39, 56%) were aged between 41–60 years. The majority of respondents were in practice for over 10 years (34/39, 87%) and were practising in adult endocrinology (34/39, 87%). Most (27/39, 69%) worked in a hospital affiliated with a university, 38% worked in a regional hospital and 28% in a private healthcare setting.

**Table 1:** Characteristics of the 39 respondents of the THESIS questionnaire:

**Respondents Characteristics**	**n (%)**
**Sex**	
Female	21 (53%)
Male	18 (47%)
**Age**	
20–40	15 (38.5%)
41–60	22 (56.4%)
> 60	2 (5.1%)
**Years in medical practice**	
< 20	21(53.8%)
21–40	17(43.6%)
> 40	1 (2.6%)
**Specialisation**	
Adult Endocrinology	34 (87.2%)
Paediatrics Endocrinology	5 (12.8%)
**Societies membership**	
IES (Irish Endocrine Society)- National society	32 (82%)
ETA (European Thyroid Association	2 (5.1%)
ATA (American Thyroid Association)	2 (5.1%)
LATS (Latin America Thyroid Association)	0 (0%)
AOTA (Asian and Oceanian Thyroid Association)	0 (0%)
Non-member	9 (23.1%)
Place of employment*	
University affiliated hospital	27 (69.2%)
Regional hospital	15 (38.5%)
Private clinic	11 (28.2%)
Specialist practice	5 (12.8%)
General practice	0
Basic researcher	0

^*^The sum of percentages exceeds 100% because some respondents were employed in more than 1 hospital

**Table 2:** LT4 formulations preferred by respondents in different clinical scenarios

	**“I expect no major changes** **with the different** **formulations”** **n (%)**	**Liquid** **solution** **n (%)**	**Tablets** **n (%)**	**Soft-gel** **capsules** **n (%)**	**Tablets form another manufacturer** **n (%)**
Interfering drugs may influence the stability of therapy. Which LT4 preparation is in your experience less likely to be subject to variable absorption?	23(58.9%)	4(10.3%)	12 (30.8%)	0 (0%)	0 (0%)
Which of the following preparations of LT4 would you prescribe in case of a first diagnosis of hypothyroidism, when the patient self-reports intolerance to various foods raising the possibility of celiac disease, malabsorption, lactose intolerance or intolerance to excipients?	9 (23.1%)	2 (5.1%)	28 (71.8%)	0 (0%)	0 (0%)
Which of the following preparations of LT4 would you prescribe for a patient established on generic LT4 who has unexplained poor biochemical control of hypothyroidism?	23(58.9%)	1(2.6%)	0 (0%)	1(2.6%)	14(35.9%)
Which of the following preparations of LT4 would you prescribe for a patient with poor biochemical control who is unable (due to busy lifestyle) to take LT4 fasting and separate from food/drink?	19 (48.7%)	2 (5.1%)	16(41%)	2 (5.1%)	0 (0%)
Which of the following preparations of LT4 would you prescribe for a patient established on generic T4 who has good biochemical control of hypothyroidism but continues to have symptoms?	31 (79.5%)	0 (0%)	0 (0%)	1(2.6%)	7 (17.9%)

Most (32/39, 82%) of the respondents were current members of IES, with smaller numbers being members of the European Thyroid Association (ETA; 2/39, 5%) and American Thyroid Association (ATA; 2/39, 5%).

Respondents indicated that they treat patients with thyroid disease daily (17/39, 43%) or weekly (21/39, 53%), with only one respondent treating such patients rarely. Over half (20/39, 51.3%) treat more than a hundred hypothyroid patients per year, 46% (18/39) treat 50–100 hypothyroid patients annually, and only one rarely managed hypothyroid patients.

### Treating hypothyroid patients

**Fig. 1:**
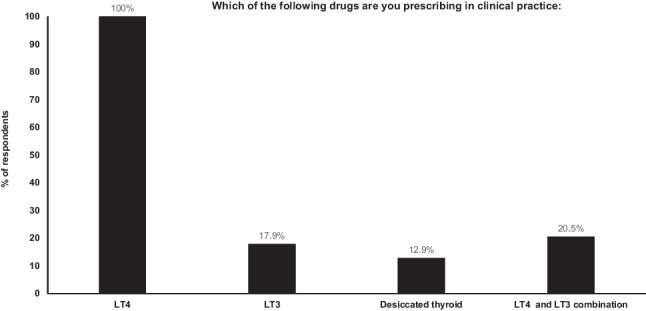
Forms of thyroid hormone used in clinical practice

All respondents (100%) stated that their treatment of choice for hypothyroidism is LT4, and all prescribed it in their clinical practice. Seven (17.9%) prescribe LT3, 8 (20.5%) prescribe LT4 + LT3 combination therapy, and five (12.9%) prescribe DTE (Fig. 1).

### Using different LT4 formulations

Forty-three per cent of respondents indicated that they have a significant influence on the management of their patients with hypothyroidism, because most of their patients were dispensed the type of LT4 they recommended. Over a third (38.4%) reported no control over the type of LT4 dispensed to their patients, and a minority (15.4%) indicated that GPs choose the type of LT4 dispensed.

Participants were asked about their preference of LT4 formulation in specific clinical circumstances (Table 2). In all scenarios, participants expressed a strong preference for LT4 tablets, and most did not expect any major changes in thyroid status with alternative formulations, such as liquid LT4 solution, soft-gel capsules, or tablets from another manufacturer. A third of participants would prescribe LT4 from an alternative manufacturer if a patient on generic LT4 had unexplained poor biochemical control of hypothyroidism.

### Monitoring thyroid hormone treatment

After initiating LT4 replacement therapy for hypothyroid patients, most (21/39, 54%) respondents would recheck serum TSH after 4–6 weeks, and the remainder (18/39, 46%) would recheck after 8 weeks.

Following a change to another LT4 formulation or manufacturer, 36% (14/39) would recheck serum TSH after 4–6 weeks, 41% (16/39) would recheck TSH after 8 weeks and 18% (7/39) stated that there is no need for TSH monitoring provided the LT4 dose remains the same. Only two respondents (5.1%) indicated that timing of repeat TSH measurement would depend on clinical evaluation.

### Treating euthyroid patients with thyroid hormones

**Fig. 2:**
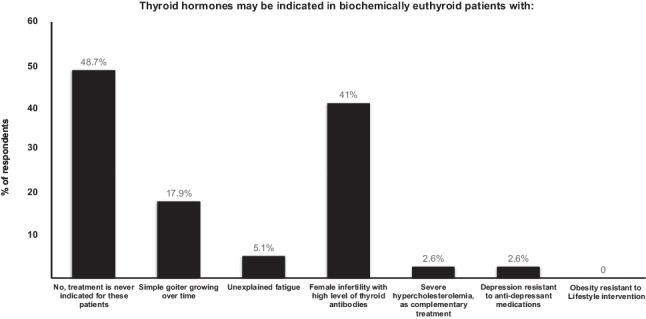
Use of thyroid hormones in different conditions

The attitude of Irish endocrinologists to prescribing thyroid hormone therapy for biochemically euthyroid patients in different clinical situations was explored (Fig. 2). Most of the respondents (19/39, 48%) stated that use of TH in such patients is never indicated in the scenarios provided, however many (16/39, 41%) would consider treating anti-thyroid autoantibody positive females with infertility. Seven endocrinologists (18%) would consider using thyroid hormone therapy in euthyroid patients with a simple goitre growing over time, 2 (5%) would consider it in euthyroid patients with unexplained fatigue. Conditions such as severe hypercholesterolemia and depression resistant to antidepressant medications were considered indications for treatment by a small number of respondents (1/39, 2% for each). None of the respondents would consider LT4 treatment in obesity resistant to lifestyle interventions.

### Combination therapy with LT4 and LT3

**Fig. 3:**
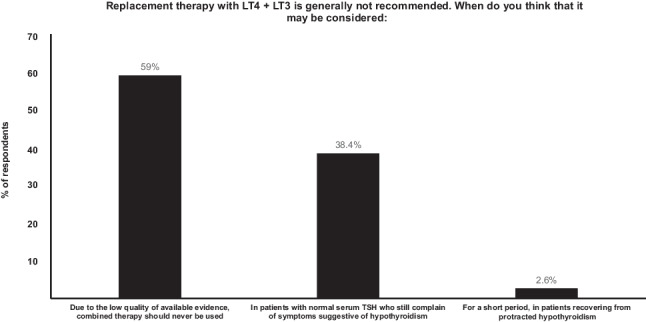
Indication for using combination LT4 + LT3 therapy

Combination treatment with LT4 and LT3 is not used by most Irish endocrinologists (23/39, 59%), however 38% (15/39) would consider it for patients on LT4 who have a normal serum TSH concentration, but persistent symptoms suggestive of hypothyroidism (Fig. 3). One respondent considered use of combination treatment in patients recovering from protracted hypothyroidism. Physicians working in university-affiliated hospitals are 2.3 times less likely to use combined replacement [4/27 (14%) vs 3/12 (25%), *p* = 0.02].

### Persistent symptoms in LT4-treated patients

The survey probed the perception of Irish endocrinologists regarding prevalence and trends of persistent hypothyroid symptoms amongst hypothyroid patients on LT4. Most respondents (14/39, 36%) estimated that 6–10% of hypothyroid patients experience persistent symptoms despite biochemical euthyroidism on LT4 therapy, 26% (10/29) estimated 11–30% and 23% (9/39) estimated fewer 5% of their patients experienced this. Only three (8%) of the respondents observe it in more than 30% of their hypothyroid patients. The remaining respondents (3/39, 8%) were not sure.

The frequency of this phenomenon was perceived to be increasing; 61% (24/39) stated that presentation of such cases has increased over the past five years. Eleven (28%) have not detected any change. Only one respondent (2.6%) reports seeing fewer cases, and the remaining 3 (7.7%) were unsure.

**Fig. 4:**
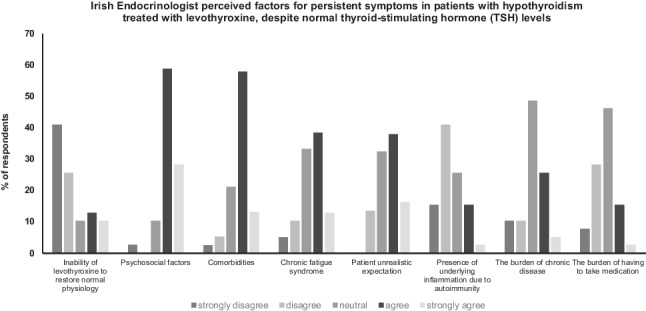
Irish endocrinologists’ speculation concerning possible factors explaining persistent symptoms of hypothyroidism despite biochemical euthyroidism in patients treated with LT4

Participants were asked about their opinion of the underlying cause of persistent hypothyroid symptoms in LT4- treated hypothyroid patients with a normal concentration of serum TSH. The majority of physicians indicated (strongly agree or agree) that key factors included psychosocial factors (34/39, 87%), comorbidities (27/38, 71%), chronic fatigue syndrome (20/39, 51%) and unrealistic patient expectations (22/39, 56%) as most likely causes for persistent symptoms. Two thirds (26/39, 67%) did not agree (strongly disagree, disagree) that symptoms may be due to the inability of levothyroxine to restore normal physiology and 56% (22/39) disagreed that the presence of underlying inflammation causes persistent symptoms (Fig. 4).

The majority of respondents (48.7%) indicated that supplementation with selenium or iodine should never be used, while 38.5% would consider their use if requested by the patient. Only 4% use selenium or iodine in patients with co-existing autoimmune thyroiditis and one respondent would consider it in patients with subclinical hypothyroidism.

Only two respondents reported having a personal diagnosis of hypothyroidism, of whom one reported excessive fatigue. Neither had tried combination LT4 and LT3 therapy or DTE. A minority of respondents (8%) reported they would try combination LT4 and LT3 therapy or DTE should they develop hypothyroidism later.

## Discussion

This survey confirms that, consistent with international guidelines, LT4 is the preferred treatment option for hypothyroidism amongst Irish endocrinologists [3, 17, 18]. Tablets were by far the preferred LT4 formulation in every clinical scenario described, however many (36%) indicated that they would change to tablets from another manufacturer in the setting of unexplained poor biochemical control. Only a minority consider liquid solution in some circumstances (use of drugs affecting absorption, possible lactose intolerance, patient inability to administer LT4 in fasting state) and very few use soft-gel capsules. This is consistent with the evidence available to date regarding alternative formulations [6], and also findings from the other THESIS surveys [16, 19–34].

Biochemical monitoring of thyroid function tests was performed by all respondents, most commonly at 4–6 weeks, as recommended by guidelines [18], but many rechecked TSH at slightly less frequent time intervals (after 8 weeks).

A number of respondents would use TH in biochemically euthyroid individuals. Almost 18% would use TH in a simple goitre that was growing over time; this is in contrast to guidance from professional organisations [35, 36], due to the ensuing TSH suppression, which is in turn associated with increased likelihood of atrial fibrillation, cardiovascular disease [37], reduced bone mineral density, fractures [38] and excessive mortality [39]. Respondents’ practices in other European countries vary considerably, but in many countries over 40% respondents indicated they would use TH in this scenario [20, 24–26], so the percentage of Irish physicians using TH in this scenario seems quite low in comparison.

Very small numbers use it in euthyroid individuals with unexplained fatigue, severe hypercholesterolaemia and depression, where there is little or no data to support the use of TH [18]. Perhaps this is not surprising when taking the global trend of increased TH use into account; a study from 2014 showed that 6% of people newly prescribed LT4 in the UK had a serum TSH within the normal range prior to treatment [40], with the equivalent figure in the USA being 28% [41].

In 2019, a large UK trial of thyroxine use in euthyroid women with positive thyroid peroxidase autoantibodies did not show an increase in live birth rate compared to placebo [42]. Despite this, many Irish endocrinologists would use TH in biochemically euthyroid women with infertility and positive anti-thyroid antibodies. This finding is similar to that seen in other THESIS surveys; overall rates of TH use in such women were high, but varied from 30 to 63% [24, 26]. International recommendations still vary slightly in this setting; a recent guideline from the ETA recommended the use of LT4 in all women seeking advice for subfertility with TSH higher than 4.0 mIU/L (or higher than the upper limit of the reference range) and suggested that LT4 could be considered on a case-by-case basis in women with positive thyroid autoantibodies and TSH level of 2.51 mU/L to 4.0 mU/L [43]. ATA guidelines from 2017 state that there is insufficient evidence to recommend LT4 therapy in euthyroid, auto-antibody positive women attempting natural conception, but LT4 could be considered in such women if undergoing assisted reproductive techniques [44]. Our survey question contains some ambiguity, since biochemical euthyroidism was not exactly defined, and subclinical hypothyroidism has been variably defined in studies related to fertility and pregnancy. In addition, the question did not specify whether the patient was planning assisted reproductive techniques. These factors may have affected the respondent’s likelihood of prescribing TH.

The majority of Irish endocrinologists never use combination LT4 and LT3 therapy, however a significant number (38%) would consider it in biochemically euthyroid patients with symptoms suggestive of hypothyroidism. This is higher than in the USA, where 3.6% would use it in a similar clinical scenario [45], but similar to other European countries, for example THESIS survey results from Finland indicated that 43% of respondents would consider combination therapy [24]. Notably, this is at variance with the available evidence. The vast majority of randomized trials demonstrated that combined T4-T3 treatment is not superior to T4 monotherapy for the improvement of hypothyroid symptoms [46]. The most commonly used LT3-containing replacement regimen was LT4 and LT3 combination therapy, although 1 in 8 use DTE, also contrary to recommendations [3].

Respondents indicated that the most common reasons for persistent symptoms in hypothyroid patients despite normalisation of TSH were psychosocial factors, comorbidities, unrealistic patient expectations, and chronic fatigue syndrome. Most disagreed with the statement that LT4 does not restore normal physiology. These responses were similar to those from other European countries where THESIS was performed [16, 19–34]. Most (61%) indicated that, in their experience, this phenomenon was relatively common (6–30%) and that they are seeing more cases; this is a greater proportion than reported in other THESIS surveys [47]. Curiously, the frequency of such symptoms has been linked to gross domestic product (GDP) of the country, with higher prevalence in countries with higher GDP [47]. It is interesting to note that Ireland had the third highest GDP worldwide in 2020 [48], so perhaps the high prevalence of symptomatology reported by Irish physicians further substantiates this association.

Irish endocrinologists do not routinely recommend dietary supplements for patients with hypothyroidism, however some (38%) would consider these at the patient’s request. Although selenium may reduce thyroid auto-antibody levels, there is no evidence that it halts progression of autoimmune thyroiditis and there may be risks associated with excessive intake, so selenium supplementation is not recommended for hypothyroid patients [49]. The use of iodine in pharmacological doses may worsen thyroid status, so iodine supplementation is also not routinely recommended [18].

Our study had a seemingly very high response rate, with almost complete data acquisition, although the caveats below need to be taken into account. It is the first such study performed in Ireland and as such provides important information on how hypothyroidism is managed in this country. Many forms of TH replacement (including liothyronine and DTE) are easily available and dispensed in Ireland, and physicians’ prescription of these is not limited in any way, so the opinions expressed reflect practice that is unlimited by access to these medications. The data obtained provides information pertaining to the opinions of endocrinologists specifically (rather than general or family physicians), because participation was limited to endocrinologists.

In comparison to colleagues who participated in other THESIS surveys in Europe, Irish physicians are more reluctant to use TH in euthyroid individuals (except in the setting of a female with positive anti-thyroid antibodies who is seeking fertility, where rates are similar), and are slightly less likely to use combination therapy with LT4 and LT3. A strong preference for TH replacement with LT4 tablets is present throughout Europe.

There are some limitations to our study. The number of respondents in our survey was lower than in THESIS surveys conducted in European countries with similar populations [23, 24]. There may be a number of reasons for this; firstly, there was a stepwise approach to recruitment, because IES members were initially asked to indicate their willingness to participate by email and were then later sent a link to questionnaire by email. This additional stage in participation may have resulted in reduced response numbers. Secondly, with only 0.9 endocrinologists per 100,000 population, Ireland has too few endocrinologists; (the recommended ratio is 1.6–1.9 per 100,000). A HSE report from 2014 stated that Ireland had only 47 endocrine consultants, so we estimate that, although small, our sample is representative of the national endocrinology opinion [50]. We acknowledge that most patients with hypothyroidism are managed in primary care however, and the survey does not reflect management of hypothyroidism amongst non-endocrinologists.

## Conclusion

Consistent with results from other THESIS surveys and international recommendations, LT4 tablets were the treatment of choice for hypothyroidism in Ireland. Other formulations of LT4 were not commonly used. A substantial number of Irish endocrinologists either regularly use, or would consider, LT3 therapy for hypothyroid patients, and a minority would give thyroid hormones to euthyroid individuals in specific circumstances, such as thyroid auto-antibody positive women with a history of infertility. Most respondents considered psychosocial factors and comorbidities the underlying explanations for persistent symptomatology in LT4 treated patients. The prescription of TH amongst Irish endocrinologists was generally in keeping with recommended practice, and areas where practice deviated from guidance was typically where evidence was conflicting or insufficient.

### Supplementary Information

Below is the link to the electronic supplementary material.

Supplementary file1 (DOCX 29 KB)

Supplementary file2 (DOCX 27 KB)

Supplementary file3 (PPTX 41 KB)

Supplementary file4 (DOCX 18 KB)

## Data Availability

Raw data can be requested by contacting the corresponding author.

## Abbreviations

ATA: American Thyroid Association
DTE: Desiccated thyroid extract
ETA: European Thyroid Association
HSE: Health Service Executive
LT3: Liothyronine
LT4: Thyroxine
TH: Thyroid hormone
THESIS: Treatment of Hypothyroidism in the Europe by Specialists: an International Survey
