# Supplementary material for: Use of Thyroid Hormones in Hypothyroid and Euthyroid Patients: A THESIS questionnaire survey of members of the Irish Endocrine Society
Source: Ir J Med Sci. 2022 Dec 8;192(5):2179–87. doi: 10.1007/s11845-022-03235-z (PMC10522726; doi:10.1007/s11845-022-03235-z)
Supplement: Supplementary file 1 — Supplementary file1 (DOCX 29 KB) [file 11845_2022_3235_MOESM1_ESM.docx]

**Supplemental Table 1**

**Brands and formulations of oral thyroid hormones available to prescribers in Ireland***

1. **Licenced**

| Thyroid hormone | Pharmaceutical  formulation | Name of product | Strength | Manufacturer |
| --- | --- | --- | --- | --- |
| Levothyroxine (LT4) | Tablets | Eltroxin® | 25 mcg | Amdipharm |
| Levothyroxine (LT4) | Tablets | Eltroxin® | 50 mcg | Amdipharm |
| Levothyroxine (LT4) | Tablets | Eltroxin® | 100 mcg | Amdipharm |
| Levothyroxine (LT4) | Tablets | Levothyroxine Teva | 25mcg | Teva |
| Levothyroxine (LT4) | Tablets | Levothyroxine Teva | 50mcg | Teva |
| Levothyroxine (LT4) | Tablets | Levothyroxine Teva | 100mcg | Teva |
| Levothyroxine (LT4) | Oral solution | Levothyroxine oral solution | 25mcg/5ml | Amdipharm |
| Levothyroxine (LT4) | Oral solution | Levothyroxine oral solution | 50mcg /5ml | Amdipharm |
| Levothyroxine (LT4) | Oral solution | Levothyroxine oral solution | 100mcg/5ml | Amdipharm |

1. **Unlicenced**

| Thyroid hormone | Pharmaceutical formulation | Name of product | Strength [µg] | | Manufacturer | |  |
| --- | --- | --- | --- | --- | --- | --- | --- |
| LT4 |  |  |  | |  | |  |
| Levothyroxine (LT4) | Tablets | L-Thyroxin Henning | 25mcg | | Sanofi | |  |
| Levothyroxine (LT4) | Tablets | L-Thyroxin Henning | 100 mcg | | Sanofi | | |
| Levothyroxine (LT4) | Tablets | L-Thyrox® Hexal® | 112 mcg | | Hexal | | |
| Levothyroxine (LT4) | Tablets | L-Thyrox® Hexal® | 88 mcg | | Hexal | | |
| Levothyroxine (LT4) | Tablets | Synthroid | 137 mcg | | Abbvie | | |
| Levothyroxine (LT4) | Suspension | Levothyroxine | 125mcg/5ml | | Target Healthcare | |  |
| Levothyroxine (LT4) | Oral solution | Levothyroxine | 50mcg/5ml | | EMP | |  |
| Levothyroxine (LT4) | Oral solution | Tirosint | 100 mcg/1ml | | IBSA | |  |
| LT3 |  |  |  | |  | |  |
| Liothyronine (LT3) | Tablets | Liothyronine | 5mcg | | Sigma | |  |
| Liothyronine (LT3) | Tablets | Thybon Henning | 20mcg | | Sanofi | |  |
| Liothyronine (LT3) | Tablets | Liothyronine | 25mcg | | Sigma | |  |
| Liothyronine (LT3) | Tablets | Thybon | 100mcg | | Sanofi | |  |
| Combination LT4 & LT3 | |  | |  | |  | |
| Levothyroxine (LT4) &  Liothyronine (LT3 | Tablets | Armour Thyroid | 15mg  (LT4 76mcg,  LT3 18mcg) | | Allergan | |  |
| Levothyroxine (LT4) &  Liothyronine (LT3 | Tablets | Armour Thyroid | 30mg  (LT4 76mcg,  LT3 18mcg) | | Allergan | |  |
| Levothyroxine (LT4) &  Liothyronine (LT3 | Tablets | Armour Thyroid | 60mg  (LT4 76mcg,  LT3 18mcg) | | Allergan | |  |
| Levothyroxine (LT4) &  Liothyronine (LT3 | Tablets | Armour Thyroid | 90mg  (LT4 76mcg,  LT3 18mcg) | | Allergan | |  |
| Levothyroxine (LT4) &  Liothyronine (LT3 | Tablets | Armour Thyroid | 120mg  (LT4 76mcg,  LT3 18mcg) | | Allergan | |  |
| Levothyroxine (LT4) &  Liothyronine (LT3 | Tablets | Novothyral | (LT4 100mcg,  LT3 20mcg) | | Merck | |  |
| Levothyroxine (LT4) &  Liothyronine (LT3 | Tablets | Euthyral | (LT4 100mcg,  LT3 20mcg) | | Acella pharmaceuticals | |  |
| Levothyroxine (LT4) &  Liothyronine (LT3 | Tablets | NP Thyroid | 15mg  (LT4 9.5mcg,  LT3 2.25mcg) | | Acella pharmaceuticals | |  |
| Levothyroxine (LT4) &  Liothyronine (LT3 | Tablets | NP Thyroid | 30mg  (LT4 19mcg,  LT3 4.5mcg) | | Acella pharmaceuticals | |  |
| Levothyroxine (LT4) &  Liothyronine (LT3 | Tablets | NP Thyroid | 60mg  (LT4 38mcg,  LT3 9mcg) | | Acella pharmaceuticals | |  |
| Levothyroxine (LT4) &  Liothyronine (LT3 | Tablets | NP Thyroid | 90mg  (LT4 57mcg,  LT3 13.5mcg) | | Acella pharmaceuticals | |  |
| Levothyroxine (LT4) &  Liothyronine (LT3 | Tablets | Nature Thyroid | 32.5mg (0.5 grain)  (LT4 19mcg,  LT3 4.5mcg) | | RLC Labs | |  |
| Levothyroxine (LT4) &  Liothyronine (LT3 | Tablets | Nature Thyroid | 48.75 mg (0.75 grain)  (LT4 28.5mcg,  T3 6.75mcg) | | RLC Labs | |  |
| Levothyroxine (LT4) &  Liothyronine (LT3 | Tablets | Nature Thyroid | 65mg (1 grain)  (LT4 38mcg,  LT3 9mcg) | | RLC Labs | |  |
| Levothyroxine (LT4) &  Liothyronine (LT3 | Tablets | Nature Thyroid | 81.25mg (1.25 grain)  (LT4 47.5mcg,  LT3 11.25mcg) | | RLC Labs | |  |
| Levothyroxine (LT4) &  Liothyronine (LT3 | Tablets | Nature Thyroid | 113.75mg (1.75 grain)  (LT4 66.5mcg,  LT3 15.75mcg) | | RLC Labs | |  |
| Levothyroxine (LT4) &  Liothyronine (LT3 | Tablets | Nature Thyroid | 130mg (2 grains)  (LT4 76mcg,  LT3 18mcg) | | RLC Labs | |  |
| Levothyroxine (LT4) &  Liothyronine (LT3 | Tablets | WP Thyroid (Westhroid) | 16.25mg (0.25 grain)  (LT4 9.5mcg,  LT3 2.25mcg) | | RLC Labs | |  |
| Levothyroxine (LT4) &  Liothyronine (LT3 | Tablets | WP Thyroid (Westhroid) | 32.5mg (0.5 grain)  (LT4 19mcg,  LT3 4.5mcg) | | RLC Labs | |  |
| Levothyroxine (LT4) &  Liothyronine (LT3 | Tablets | WP Thyroid (Westhroid) | 65g (1 grain)  (LT4 38mcg,  LT3 9mcg) | | RLC Labs | |  |

*Information correct as of 19^th^ April 2022
