# Supplementary material for: Use of Thyroid Hormones in Hypothyroid and Euthyroid Patients: A THESIS questionnaire survey of members of the Irish Endocrine Society
Source: Ir J Med Sci. 2022 Dec 8;192(5):2179–87. doi: 10.1007/s11845-022-03235-z (PMC10522726; doi:10.1007/s11845-022-03235-z)
Supplement: Supplementary file 2 — Supplementary file2 (DOCX 27 KB) [file 11845_2022_3235_MOESM2_ESM.docx]

**Supplemental Table 2**

**THESIS SURVEY**

**Section A**

**A: ABOUT YOU**

**A1. Sex**

- 1. Female
  2. Male

**A2. Age** (years) *[dropdown menu]*

- 1. 20-30
  2. 31-40
  3. 41-50
  4. 51-60
  5. 61-70
  6. 70+

**A3. Years in medical practice** *[dropdown menu]*

- 1. 0-10
  2. 11-20
  3. 21-30
  4. 31-40
  5. More than 40

**A4. Specialty** [*check all that apply]*

- 1. Endocrinology
  2. Internal Medicine
  3. Pediatric Endocrinology
  4. Nuclear Medicine
  5. Surgery
  6. Family Medicine
  7. Gynecology
  8. Other

**A5. Member of…** [*check all that apply]*

- 1. ETA (European Thyroid Association)
  2. ATA (American Thyroid Association)
  3. LATS (Latin American Thyroid Association)
  4. AOTA (Asian and Oceanian Thyroid Association)
  5. National Endocrine Societies
  6. None of the above

**A6. Where do you practice?** *[check all that apply]*

- 1. University centre
  2. Regional hospital
  3. Private clinic
  4. General Practice
  5. Basic researcher
  6. Specialist Practice

**A7. Do you treat thyroid patients on a regular basis (daily or weekly)?**

- 1. Yes, daily
  2. Yes, weekly
  3. No, I rarely treat thyroid patients

**A8. Do you treat patients with hypothyroidism?**

1. Yes, from 10 to 50 patients/year
2. Yes, from 51 to 100 patients/year
3. Yes, > 100 patients/year
4. No, I rarely treat hypothyroid patients

**Section B**

**B. HYPOTHYROIDISM**

**B1. Thyroid hormones may be indicated in biochemically euthyroid patients with:** *[check all that apply]*

1. unexplained fatigue
2. obesity resistant to life-style interventions
3. severe hypercholesterolemia, as a complementary treatment
4. depression resistant to anti-depressant medications
5. female infertility with high level of thyroid antibodies
6. simple goiter growing over time*
7. no, treatment is never indicated for these patients

**If this is ever an indication, even under specific circumstances (e.g. taking into account age and comorbidities) then it should be ticked.*

**B2. Which thyroid hormones available for substitution therapy should be the first choice for the treatment of hypothyroid patients?**

1. LT4
2. LT3
3. Desiccated thyroid
4. LT4 and LT3 combination

**B3. Which of the following drugs are you prescribing in clinical practice?** *[check all that apply]*

1. LT4
2. LT3
3. Desiccated thyroid
4. LT4 and LT3 combination

**B4. How much control do you have over the formulation of LT4 dispensed for your patients? Please choose the option the best applies to your practice**

1) Most of my patients are dispensed the type of LT4 that I recommend

2) I have control over the type of LT4, but I have to justify it to the regulatory authorities every time I recommend it

3) the type of dispensed thyroxine is mostly chosen by general practitioners

4) for most of my patients I have no control over the type of LT4 that they are dispensed

**B5. Interfering drugs may influence the stability of therapy. Which LT4 preparation is in your experience least likely to be subject to variable absorption?**

1) Tablets

2) Soft-gel capsules

3) Liquid solution

4) I expect no major changes with different formulations

**B6. Which of the following preparations of LT4 would you prescribe in case of first diagnosis of hypothyroidism when the patient self-reports intolerance to various foods raising the possibility of celiac disease, malabsorption, lactose intolerance, or intolerance to common excipients?**

1) Tablets

2) Soft-gel capsules

3) Liquid solution

4) I expect no major changes with the different formulations

**B7. Which of the following preparations of LT4 would you prescribe for a patient established on LT4 who has unexplained poor biochemical control of hypothyroidism?**

1) Tablets

2) Soft-gel capsules

3) Liquid solution

4) I expect no major changes with the different formulations

**B8. Which of the following preparations of LT4 would you prescribe for a patient with poor biochemical control who is unable (due to busy lifestyle) to take LT4 fasted and separate from food/drink?**

1) Tablets

2) Soft-gel capsules

3) Liquid solution

4) I expect no major changes with the different formulations

**B9. Which of the following preparations of LT4 would you prescribe for a patient established on LT4 tablets who has good biochemical control of hypothyroidism but continues to have symptoms?**

1) Tablets

2) Soft-gel capsules

3) Liquid solution

4) I expect no major changes with the different formulations

**B10. After the start of LT4 replacement therapy, when would you re-check serum TSH:**

1. After 2 weeks
2. After 4 – 6 weeks
3. After 8 weeks
4. No, I mostly rely on clinical evaluation

**B11. In case of a switch to a different formulation or change from one manufacturer’s LT4 tablet to another, when do you recommend that the serum TSH should be re-checked:**

1. after 4 to 6 weeks
2. after 8 weeks
3. on the basis of clinical evaluation
4. no, there is no need of TSH control after preparation changes if the dosage is the same

**B12. Dietary supplements (such as selenium or iodine) are proposed for patients with thyroid disease. Do you think that they may be used in addition to thyroid hormone replacement in hypothyroidism?**

1) When there is coexisting autoimmune thyroiditis

2) In subclinical hypothyroidism

3) At the patient’s request or as a complementary treatment

4) No, dietary supplements should never be used

**B13. The use of combined replacement therapy, with administration of both LT4 and LT3, is generally not recommended. Do you think that may be considered:**

1. For a short period, in patients recovering from protracted hypothyroidism
2. In patients with normal serum TSH who still complain of symptoms suggestive of hypothyroidism
3. In hypothyroid patients with normal serum TSH who complain of unexplained weight gain
4. Due to the low quality of available evidence, combined therapy should never be used.

**B14. It has been reported that some patients with hypothyroidism treated with levothyroxine continue to experience persistent symptoms despite normal serum TSH. The following three questions refer to such patients.**

**In your clinical practice how common is this phenomenon?**

1. less than 5% of patients
2. 6-10%
3. 11-30%
4. More than 30%
5. Not sure

**B15. It has been reported that some patients with hypothyroidism treated with levothyroxine continue to experience persistent symptoms despite normal serum TSH.**

**In your experience what has been the trend over the past 5 years?**

1. I am seeing more such cases
2. I am seeing fewer such cases
3. No change
4. Not sure

**B16. In most patients treated with levothyroxine who achieve normal serum TSH, persistent symptoms are due to:**

1. inability of levothyroxine to restore normal physiology

*strongly disagree/disagree/neutral/agree/strongly agree*

1. psychosocial factors

*strongly disagree/disagree/neutral/agree/strongly agree*

1. comorbidities

*strongly disagree/disagree/neutral/agree/strongly agree*

1. chronic fatigue syndrome

*strongly disagree/disagree/neutral/agree/strongly agree*

1. patient unrealistic expectation

*strongly disagree/disagree/neutral/agree/strongly agree*

1. presence of underlying inflammation due to autoimmunity

*strongly disagree/disagree/neutral/agree/strongly agree*

1. the burden of chronic disease

*strongly disagree/disagree/neutral/agree/strongly agree*

1. the burden of having to take medication

*strongly disagree/disagree/neutral/agree/strongly agree*

**B17. Using your experience with patients treated with levothyroxine who achieve normal serum TSH, but continue to experience symptoms like fatigue, please rank them from 1-8, where 1 is the most likely and 8 the least likely explanation in your opinion.** *[Attribute a score from 1 (most likely) to 8 (least likely) to each item.]*

Rank (1-8)

1. the burden of having to take medication 
2. patient unrealistic expectations 
3. inability of levothyroxine to restore normal physiology 
4. psychosocial factors 
5. presence of underlying inflammation due to autoimmunity 
6. comorbidities 
7. chronic fatigue syndrome 
8. the burden of chronic disease 

**B18. Do you, yourself have a diagnosis of hypothyroidism requiring thyroid hormone treatment?**

1. Yes
2. No

**B19**. *(it will appear only in respondents who answered “yes” to question B18)* **Do you experience excessive tiredness/fatigue?**

1. Yes
2. No

**B20.** *(it will appear only in respondents who answered “yes” to question B18)* **Have you tried L-T4 and L-T3 combination treatment?**

1. Yes
2. No

**B21**. *(it will appear only in respondents who answered “yes” to question B18)* **Have you tried desiccated thyroid treatment?**

1. Yes
2. No

**B22.** *(it will appear only in respondents who answered “yes” to question B20 or B21)* If you have tried of L-T4 and L-T3 combination treatment or desiccated thyroid, please describe your experience (e.g. how effective compared with L-T4 monotherapy, whether you continue to take it, side-effects, long-term concerns).

(Space for free text)

**B23.** *(it will appear only in respondents who answered “No” to question B18)* Would you consider L-T4 and L-T3 combination treatment or desiccated thyroid for yourself if you were to develop hypothyroidism?

1. Yes
2. No

**B24. Please add comments (e.g. why you would or would not choose to take L-T4 and L-T3 combination treatment or desiccated thyroid for yourself)**

(Space for free text)
