# Supplementary material for: Use of Thyroid Hormones in Hypothyroid and Euthyroid Patients: A THESIS questionnaire survey of members of the Irish Endocrine Society
Source: Ir J Med Sci. 2022 Dec 8;192(5):2179–87. doi: 10.1007/s11845-022-03235-z (PMC10522726; doi:10.1007/s11845-022-03235-z)
Supplement: Supplementary file 3 — Supplementary file3 (PPTX 41 KB) [file 11845_2022_3235_MOESM3_ESM.pptx]

## Slide 1
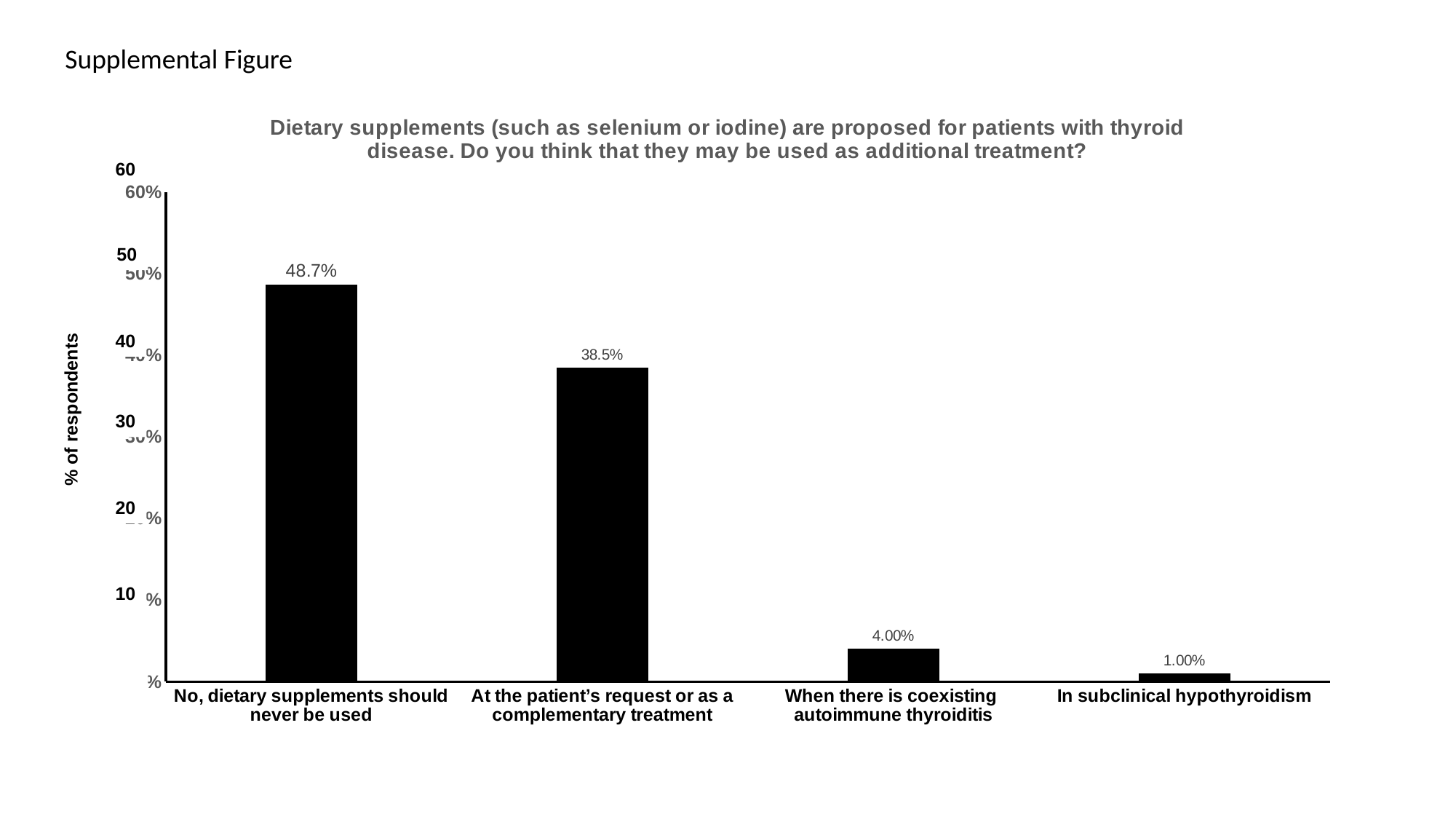

Supplemental Figure
### Chart: Dietary supplements (such as selenium or iodine) are proposed for patients with thyroid disease. Do you think that they may be used as additional treatment?
| Category | |
|---|---|
| No, dietary supplements should never be used | 0.487 |
| At the patient’s request or as a complementary treatment | 0.385 |
| When there is coexisting autoimmune thyroiditis | 0.04 |
| In subclinical hypothyroidism | 0.01 |60
50
40
% of respondents
30
20
10
10
