# Supplementary material for: Use of Thyroid Hormones in Hypothyroid and Euthyroid Patients: A THESIS questionnaire survey of members of the Irish Endocrine Society
Source: Ir J Med Sci. 2022 Dec 8;192(5):2179–87. doi: 10.1007/s11845-022-03235-z (PMC10522726; doi:10.1007/s11845-022-03235-z)
Supplement: Supplementary file 4 — Supplementary file4 (DOCX 18 KB) [file 11845_2022_3235_MOESM4_ESM.docx]

**Supplemental Figure 1: Use of Dietary Supplements in thyroid patients:**

**Figure legend**

Supplemental Figure 1. Recommendations for use of dietary supplements in patients with thyroid disease, as indicated by respondents.
